# Periarticular Infiltration Compared to Single Femoral Nerve Block in Total Knee Arthroplasty: A Prospective Randomized Study

**DOI:** 10.1055/s-0044-1785449

**Published:** 2024-04-10

**Authors:** Osmar Valadão Lopes Júnior, Juliano Munhoz Viana, Juliany Aguirre de Carvalho, Bruno Lunardi Folle, Vinícius Canelo Kuhn, Paulo Renato Fernandes Saggin

**Affiliations:** 1 Instituto de Ortopedia e Traumatologia de Passo Fundo, Passo Fundo, RS, Brasil; 2 Departamento de Ortopedia e Traumatologia, Hospital São Vicente de Paulo, Universidade Federal da Fronteira Sul, Passo Fundo, RS, Brasil

**Keywords:** analgesia, anesthetics, local, arthroplasty, replacement, knee, nerve block, inflitration, femoral nerve

## Abstract

**Objective** To compare patients undergoing total knee arthroplasty (TKA) under spinal anesthesia and single femoral nerve block (FNB) with subjects undergoing TKA under spinal anesthesia and periarticular infiltration (PAI).

**Materials and Methods** A total of 100 patients undergoing primary TKA were randomized into two groups. Group 1 included patients undergoing surgery under FNB associated with spinal anesthesia, while group 2 included patients undergoing TKA under IPA and spinal anesthesia. The assessment of these subjects in the early postoperative period included pain, active flexion, active extension, elevation of the extended limb, and morphine use.

**Results** There was no significant difference in the types of analgesia concerning pain, the elevation of the extended limb, and morphine use. Active flexion and extension were better in the PAI group ( *p* = 0.04 and *p* = 0.02 respectively).

**Conclusion** We conclude that the techniques are similar regarding pain control, limb elevation, and morphine use. The use of IPA provided better active flexion and extension during the hospital stay compared to single FNB in patients undergoing TKA.

## Introduction

More than 80% of patients who undergo surgical procedures experience acute postoperative pain, and approximately 75% of them classify this pain as moderate or severe. Evidence suggests that postoperative pain treatment is often inadequate, with direct effects on quality of life, recovery, and the risk of postoperative complications, such as chronic pain. 1 2 3 4

Among the methods to control pain after total knee arthroplasty (TKA), femoral nerve block (FNB) using a catheter and intermittent anesthetic infusion provides good postoperative analgesia. However, its potential disadvantages include a decrease in quadriceps activation, resulting in lower muscle control during gait and increased risk of falls. Recent studies 5 6 7 8 9 10 11 12 have demonstrated that intraoperative periarticular infiltration (PAI), using a cocktail of medications, produces an analgesic effect similar to that of FNB, with the advantage of not compromising muscle function.

The present study aimed to evaluate and compare patients undergoing TKA under spinal anesthesia and single FNB to patients undergoing the same procedure but under spinal anesthesia and PAI. The assessment of these subjects in the early postoperative period included pain, knee range of motion (ROM), ability to lift the limb, morphine requirement, and complications. We hypothesize that PAI produces the same analgesic effect in the early postoperative period of TKA compared to FNB, but with a lesser impact on muscle function in the initial rehabilitation phase.

## Materials and Methods

In total, 100 patients with an indication for TKA were initially selected and randomized into two groups. Group 1 included 50 patients who underwent TKA under spinal anesthesia and simple (single) FNB, and group 2 consisted of 50 patients who underwent the same surgical procedure but under spinal anesthesia and PAI.

The inclusion criteria were patients of both sexes, aged 18 or older, and undergoing primary TKA for advanced osteoarthrosis. The exclusion criteria were patients with allergies to any of the medications included in the research protocol, contraindication to or failure of spinal anesthesia, known abusers of alcohol or drugs, with rheumatoid arthritis or other inflammatory diseases, those submitted to previous surgeries (except for meniscal and ligament injury treatment), those with psychiatric illnesses diagnosed or under treatment, paralysis, paresis, or paresthesia in the contralateral limb, and patients who did not agree to participate in the study.

The participants were initially randomized into 2 groups of interest using a sequence of random numbers ordered from 1 to 100 per their entry date into the study. A responsible person kept the randomization list confidential and informed the surgeon and anesthetist to which group the patient belonged before anesthetic induction. This same person did not participate in any other phase of the study, having no contact with patients or evaluators. A team member, a doctor duly trained and blinded to the randomization, performed data collection.

Group 1 underwent spinal anesthesia with 15 mg of bupivacaine and 100 mcg of morphine; immediately after, the anesthetist performed a single FNB with 150 mg of ropivacaine and 150 mcg of clonidine aided by a peripheral nerve stimulator (Stimuplex B. Braun Medical Inc., Melsungen, Hesse, Germany). Group 2 received the same spinal anesthesia as group 1 and PAI with an analgesic solution consisting of 150 mcg of clonidine, 30 mg of ketorolac, 375 mg of ropivacaine, and 1 mg of epinephrine diluted in 50 mL of saline solution. The surgeon performed PAI during the procedure, administering 20 mL in the posteromedial capsular region, 15 mL in the posterolateral region, and distributing the remaining volume throughout the femoral and tibial subperiosteal regions (lateral, medial, and anterior).

The postoperative assessment followed a logical order, starting with pain, active and passive ROM, the active elevation of the limb, and the amount of morphine used. Patient evaluation occurred during hospitalization at 24 (first postoperative day [1PO]), 48 (2PO), and 72 (3PO) hours postoperatively. In the postoperative period, all patients underwent the same physical therapy protocol and received the same analgesia protocol, which included continuous use of 30 mg of codeine every 6 hours, 750 mg of paracetamol every 8 hours, 1 g of dipyrone every 6 hours, and 30 mg of ketorolac every 8 hours for 48 hours. The prescription of morphine at a dose of 0.05 mg/kg every 3 hours was left to the patient's discretion as needed.

Pain was assessed through the Visual Analogue Scale (VAS) as rated by the patient from 0 to 10, with 0 indicating no pain and 10, the most intense pain. The passive and active ROM were determined in degrees using a universal goniometer, with the patient in the supine position. Elevation of the extended limb is the active elevation distance in centimeters of the limb measured from the calcaneus to the physical examination table. Morphine use was quantified in mg/day and subsequently evaluated in the medical record analysis.

The study occurred from September 2019 to February 2021, and all patients underwent treatment and surgery using the same surgical technique and implants. The same anesthesia team performed the anesthetic procedure. The Teaching and Research Committee of Universidade de Passo Fundo approved the study in August 2019 (under opinion 3.537.0920), and all patients included signed the informed consent form (ICF).

### Statistical Analysis

The sample size calculation was made using the Windows Programs for Epidemiologists (Winpepi, freeware) software, version 11.65 and based on a study by Zhang et al. 7 This calculation reached a minimum total of 44 patients per group considering a 5% significance level, 80% power, and an effect size of at least 0.6 standard deviation (SD) between groups regarding the pain score.

The quantitative and ordinal variables were expressed as mean ± SD values, and the categorical variables were expressed as absolute and relative frequencies. The Shapiro-Wilk test determined data normality. Group comparison over time used the generalized estimating equations (GEE) model complemented by the least significant difference (LSD) test. The linear model was applied to variables with normal distribution, while the gamma model was used for variables with asymmetric or ordinal distribution.

The significance level adopted was of 5% ( *p* < 0.05), and the analyses employed the IBM SPSS Statistics for Windows (IBM Corp., Armonk, NY, United States) software, version 28.0.

## Results

The study included and analyzed 100 patients. Table 1 characterizes the patient sample, and the groups were similar regarding age, gender, and operated side. The average hospital stay was of four days for both groups.

**Table 1:** Sample characterization

Variables	Total sample (n = 100)	FNB group (n = 50)	PAI group (n = 50)	*p*
Age (years): mean ± SD	67.9 ± 7.7	68.1 ± 7.5	67.7 ± 7.9	0.400*
Sex: n (%)				1.000**
Male	23 (23.0)	11 (22.0)	12 (24.0)	
Female	77 (77.0)	39 (78.0)	38 (76.0)	
Location: n (%)				1.000**
Headquarters	69 (69.0)	35 (70.0)	34 (68.0)	
Branch	31 (31.0)	15 (30.0)	16 (32.0)	
Side: n (%)				0.316**
Right	54 (54.0)	30 (60.0)	24 (48.0)	
Left	46 (46.0)	20 (40.0)	26 (52.0)	
Hospitalization time (days): mean ± SD	4.28 ± 0.52	4.39 ± 0.53	4.18 ± 0.48	0.208*

**Abbreviations:** FNB, femoral nerve block; PAI, periarticular infiltration; SD, standard deviation.

**Notes:** *Student's *t* -test; **Pearson Chi-squared test.

There was no statistically significant difference in pain between the groups, neither were there differences in the interaction effect between group and time regarding pain levels. Both groups presented a significantly lower pain level on 3PO compared to 1PO and 2PO, which do not differ significantly from each other. Even after adjustment per daily morphine intake, the outcomes remained similar between the groups ( Table 2 ).

**Table 2:** Group comparison of the variables analyzed 1, 2, and 3 days after surgery

Variables	FNB group (n = 50)	PAI group (n = 50)	*p* ^#^
**Pain (VAS): mean ± SD**			
1 ^st^ day PO	4.94 ± 2.25 ^b^	4.94 ± 2.40 ^b^	1.000
2 ^nd^ day PO	4.86 ± 2.22 ^b^	4.32 ± 1.98 ^b^	0.195
3 ^rd^ day PO	2.84 ± 1.80 ^a^	2.88 ± 1.99 ^a^	0.915
Difference between the 1 ^st^ and 3 ^rd^ days PO (95%CI)	−2.10 (−2.68 to −1.52)	−2.06 (−2.69 to −1.43)	0.199**
**Active ROM in flexion: mean ± SD**			
1 ^st^ day PO	42.7 ± 22.4 ^a^	47.9 ± 22.9 ^a^	0.248
2 ^nd^ day PO	56.2 ± 18.8 ^b^	63.4 ± 17.4 ^b^	**0.046**
3 ^rd^ day PO	77.8 ± 15.1 ^c^	83.0 ± 11.2 ^c^	**0.047**
Difference between the 1 ^st^ and 3 ^rd^ days PO (95%CI)	35.1 (29.5 to 40.6)	35.1 (29.8 to 40.4)	0.571**
**Active ROM in extension: mean ± SD**			
1 ^st^ day PO	1.30 ± 2.44 ^b^	1.22 ± 2.17 ^b^	0.869
2 ^nd^ day PO	1.10 ± 2.32 ^ab^	0.50 ± 1.52 ^a^	0.122
3 ^rd^ day PO	0.80 ± 1.85 ^a^	0.80 ± 1.85 ^ab^	1.000
Difference between the 1 ^st^ and 2 ^nd^ days PO (95%CI)	−0.20 (−0.47 to 0.07)	−0.72 (−1.21 to −0.24)	0.025**
Difference between the 2 ^nd^ and 3 ^rd^ days PO (95%CI)	−0.30 (−0.73 to 0.13)	0.30 (−0.13 to 0.73)	
Difference between the 1 ^st^ and 3 ^rd^ days PO (95%CI)	−0.50 (−1.00 to −0.00)	−0.42 (−1.10 to 0.25)	
**Elevation (cm): mean ± SD**			
1 ^st^ day PO	16.9 ± 21.8 ^a^	22.7 ± 25.4 ^a^	0.222
2 ^nd^ day PO	26.6 ± 24.2 ^b^	35.2 ± 25.0 ^b^	0.078
3 ^rd^ day PO	39.4 ± 23.6 ^c^	43.3 ± 23.7 ^c^	0.401
Difference between the 1 ^st^ and 3 ^rd^ days PO (95%CI)	22.5 (18.1 to 26.9)	20.7 (15.4 to 26.0)	0.160**
**Morphine use (mg)**			
1 ^st^ day PO	3.62 ± 3.95 ^b^	4.20 ± 4.51 ^b^	0.489
2 ^nd^ day PO	2.71 ± 3.71 ^b^	2.95 ± 3.08 ^b^	0.722
3 ^rd^ day PO	0.59 ± 1.39 ^a^	1.16 ± 2.87 ^a^	0.201
Difference between the 1 ^st^ and 3 ^rd^ days PO (95%CI)	−3.03 (−4.02 to −2.04)	−3.04 (−4.48 to −1.60)	0.880**

**Abbreviations:** 95%CI, 95% confidence interval; FNB, femoral nerve block; PAI, periarticular infiltration; PO, postoperatively; ROM, range of motion; SD, standard deviation; VAS, Visual Analog Scale.

**Notes:** *+ = 1/++ = 2/+++ = 3/++++ = 4; ** group versus time interaction effect; ^a,b,c^ Intragroup comparison: equal letters denote lack of difference per the least significant difference (LSD) test at 5% significance; ^#^ comparison between the groups using the LSD test and the generalized estimating equations (GEE) model.

**Fig. 1:**
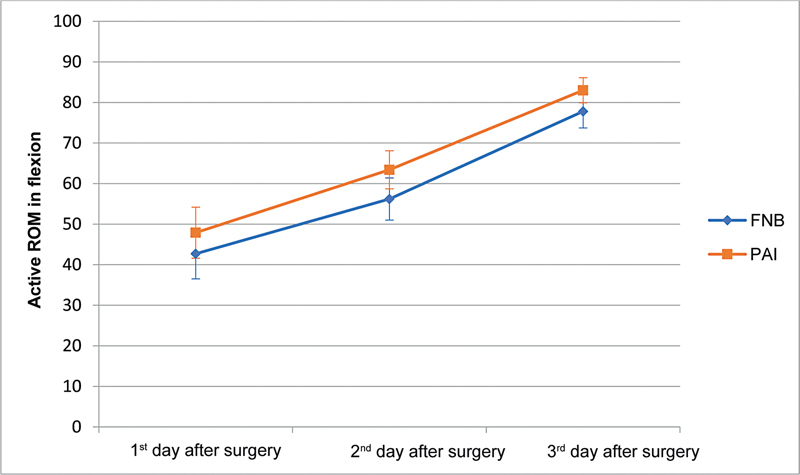
Assessment of the active range of motion (ROM) in flexion per time and study group. FNB, femoral nerve block; PAI, periarticular infiltration.

**Fig. 2:**
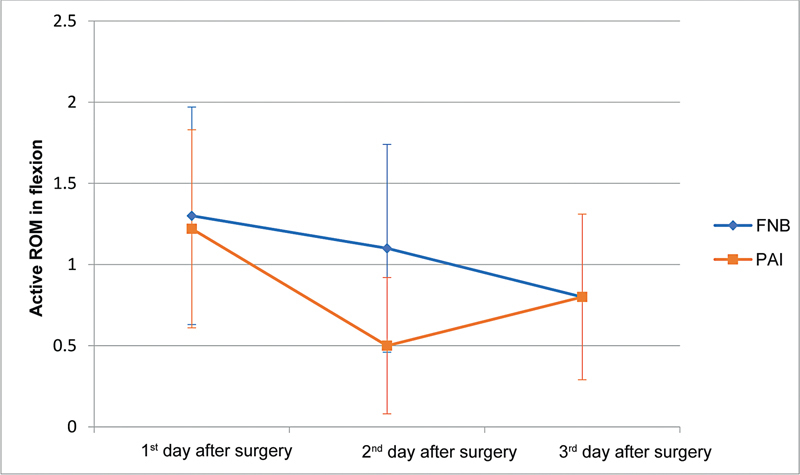
Assessment of the active ROM in extension per time and study group. FNB, femoral nerve block; PAI, periarticular infiltration.

The ROM presented a statistically significant difference between the groups: the PAI group presented significantly higher mean active flexion on 2PO ( *p* = 0.046) and 3PO ( *p* = 0.047) when compared to the FNB group ( Fig. 1 ). Active extension was significantly different over time between the groups (significant interaction effect; *p* = 0.025): the PAI group presented a significant reduction on 2PO (with an average value of 0.72), and the FNB group presented a significant decrease only on 3PO ( Fig. 2 ). However, despite the statistical difference between the groups, we believe it may not be clinically relevant in the medium and long terms.

Active flexion, hip contraction, and elevation parameters increased significantly from 2PO onwards in both groups. Morphine use only decreased significantly on 3PO in both groups.

Two patients (one from each group) had allergic reactions: itching and rash; and one patient (from the FNB group) fell to the ground from their own height on 2PO, with no other consequences.

## Discussion

Several studies have demonstrated that FNB and PAI are reliable techniques to control pain in the early TKA postoperative period. 5 10 Inadequate pain control in the TKA postoperative period increases the risk of chronic pain, resulting in lower quality of life, prolonged hospital stay, and increased treatment costs. 13 14 15

The present study aimed to compare a single FNB with PAI in the early TKA postoperative period. We confirmed the hypothesis of adequate pain control associated with better muscle contraction capacity during the early postoperative phase in the PAI group. The single FNB group presented a deleterious effect on muscle function evidenced by active flexion and extension during the initial rehabilitation phase, consistent with the suggestion by some authors. 5 7 16

The primary sensory innervation of the knee comes from the femoral nerve anteriorly and the posterior cutaneous nerve of the thigh posteriorly. Single FNB or continuous analgesia with a catheter are postoperative analgesia methods that provide better pain control and reduce opioid consumption and its adverse effects. 12 However, it is worth highlighting that regional anesthetic techniques require a specialist in anesthesia, present a failure rate of 0% to 67%, and the risk of falls due to motor inhibition has been reported by some authors, 16 17 18 19 20 21 22 mainly with the use of blocks with a continuous anesthetic release via a catheter. In the present study, a patient from the FNB group fell on 2PO. Although the patient did not present any major motor limitations during the evaluation, we cannot exclude the FNB as a causal factor.

In the clinical practice, PAI gained prominence due to the fact that several studies have shown outcomes comparable to those of FNB in controlling pain and opioid use. 7 8 9 11 12 In a systematic review of 14 randomized clinical trials, Albrecht et al. 5 demonstrated pain relief and similar postoperative functional outcomes between the FNB and PAI techniques. Wall et al. 10 randomized 230 patients with an indication for TKA and found no statistical difference between groups undergoing PAI and FNB. In this study, 10 it is worth highlighting that the anesthetic techniques were performed by 59 anesthetists and 33 surgeons, representing high variability in execution but preserving the reproducibility of the outcomes.

Regarding morphine use during hospital stay, some prospective studies 7 8 12 did not find significant differences. In contrast, Parvataneni et al. 11 observed divergent data, showing a greater morphine intake on 1PO in patients undergoing PAI. In the present study, subjects from both groups showed a gradual decrease in morphine use throughout the days, and there was no difference between the groups.

Regarding knee mobility, we found a significant active mobility gain in subjects from the PAI group. Berninger et al. 9 and Wall et al. 10 observed a greater flexion capacity in patients undergoing PAI, more evident on 1PO and 2PO, respectively.

Despite the difference in active mobility, in the present study we did not find significant differences between the groups regarding lower limb elevation. We believe that the lack of difference may be associated with the performance of a single FNB, that is, with no continuous anesthetic infusion. This result is not consistent with those of Parvataneni et al., 11 who found a better ability to lift the extended limb on 1PO in the PAI group and similar pain scores during the postoperative hospitalization, suggesting that PAI provides pain control equivalent to that of FNB while maintaining the motor strength of the quadriceps.

Recently, some studies evaluated the adductor canal block (ACB) and compared it with FNB and the association of ACB and PAI. The ACB resulted in outcomes similar to those of FNB in terms of analgesia control, but with less motor involvement than the FNB. 23 24 25 Regarding the association of ACB and PAI, Goytizolo et al. 26 reported no difference in the addition of ACB to PAI alone. However, further studies are required to elucidate this issue.

We believe that the present study has certain critical points. All patients were operated on by the same surgical and anesthetic teams, receiving the same protocol of PAI, FNB, and analgesic medication. To keep the procedure more reproducible in locations with no ultrasound-assisted FNB available, we opted for the single FNB technique, since the continuous infusion technique requires specific training and appropriate equipment. We did not use medium and long-term functional scores or quality of life and mental health assessment scores. We must highlight that the lack of specific studies with the same group of patients in the literature limits the precision of sample size calculation. As such, the sample size calculation may be undersized, as the only reference variable was pain. Thus, even though our results are consistent with those of the literature, we highlight the need for future studies for better procedural standardization, to investigate the effect of combining procedures, and to assess the clinical relevance of the findings.

## Conclusion

We concluded that pain control, limb elevation, and morphine use were similar between analgesia techniques. The PAI technique provided greater capacity for active knee extension and flexion during the first three postoperative days.

## References

[1] Chou R Gordon D B de Leon-Casasola O A Management of Postoperative Pain: A Clinical Practice Guideline From the American Pain Society, the American Society of Regional Anesthesia and Pain Medicine, and the American Society of Anesthesiologists' Committee on Regional Anesthesia, Executive Committee, and Administrative Council J Pain 2016 17 02 131 157 26827847 10.1016/j.jpain.2015.12.008

[2] Berghmans D DP Lenssen A F Emans P J de Bie R A Functions, disabilities and perceived health in the first year after total knee arthroplasty; a prospective cohort study BMC Musculoskelet Disord 2018 19 01 250 30045710 10.1186/s12891-018-2159-7 PMC6060557

[3] Terkawi A S Mavridis D Sessler D I Pain management modalities after total knee arthroplasty Anesthesiology 2017 126 05 923 937 10.1097/ALN.0000000000001607 28288050

[4] Ferreira M C Oliveira J CP Zidan F F Franciozi C EDS Luzo M VM Abdalla R J Total knee and hip arthroplasty: the reality of assistance in Brazilian public health care Rev Bras Ortop 2018 53 04 432 440 30027075 10.1016/j.rboe.2018.05.002 PMC6052187

[5] Albrecht E Guyen O Jacot-Guillarmod A Kirkham K R The analgesic efficacy of local infiltration analgesia vs femoral nerve block after total knee arthroplasty: a systematic review and meta-analysis Br J Anaesth 2016 116 05 597 609 10.1093/bja/aew099 27106963

[6] Dalury D F Lieberman J R MacDonald S J Current and innovative pain management techniques in total knee arthroplasty J Bone Joint Surg Am 2011 93 20 1938 1943 22012532 10.2106/JBJS.9320icl

[7] Zhang L K Ma J X Kuang M J Ma X L Comparision of periarticular local infiltration analgesia with femoral nerve block for total knee arthroplasty: a meta-analysis of randomized controlled trials J Arthroplasty 2018 33 06 1972 1.978E7 29455938 10.1016/j.arth.2017.12.042

[8] Wang C Cai X Z Yan S G Comparison of periarticular multimodal drug injection and femoral nerve block for postoperative pain management in total knee arthroplasty: a systematic review and meta-analysis J Arthroplasty 2015 30 07 1281 1286 25735501 10.1016/j.arth.2015.02.005

[9] Berninger M T Friederichs J Leidinger W Effect of local infiltration analgesia, peripheral nerve blocks, general and spinal anesthesia on early functional recovery and pain control in total knee arthroplasty BMC Musculoskelet Disord 2018 19 01 232 30021587 10.1186/s12891-018-2154-z PMC6052689

[10] P. D. H. Wall on behalf of A. P. Sprowson,† M. L. Costa, PAKA Study Group Wall P DH Parsons N R Parsons H A pragmatic randomised controlled trial comparing the efficacy of a femoral nerve block and periarticular infiltration for early pain relief following total knee arthroplasty Bone Joint J 2017 99-B 07 904 911 28663395 10.1302/0301-620X.99B7.BJJ-2016-0767.R2 PMC5633832

[11] Parvataneni H K Shah V P Howard H Cole N Ranawat A S Ranawat C S Controlling pain after total hip and knee arthroplasty using a multimodal protocol with local periarticular injections: a prospective randomized study J Arthroplasty 2007 22(6, Suppl 2)33 38 17823012 10.1016/j.arth.2007.03.034

[12] Chan E Y Fransen M Parker D A Assam P N Chua N Femoral nerve blocks for acute postoperative pain after knee replacement surgery Cochrane Database Syst Rev 2014 2014 05 CD009941 24825360 10.1002/14651858.CD009941.pub2 PMC7173746

[13] Garimella V Cellini C Postoperative pain control Clin Colon Rectal Surg 2013 26 03 191 196 24436674 10.1055/s-0033-1351138 PMC3747287

[14] Elmallah R K Cherian J J Pierce T P Jauregui J J Harwin S F Mont M A New and common perioperative pain management techniques in total knee arthroplasty J Knee Surg 2016 29 02 169 178 25892004 10.1055/s-0035-1549027

[15] American Society of Anesthesiologists Task Force on Acute Pain Management Practice guidelines for acute pain management in the perioperative setting: an updated report by the American Society of Anesthesiologists Task Force on Acute Pain Management Anesthesiology 2004 100 06 1573 1581 10.1097/00000542-200406000-00033 15166580

[16] Wick E C Grant M C Wu C L Postoperative multimodal analgesia pain management with nonopioid analgesics and techniques: a review JAMA Surg 2017 152 07 691 697 28564673 10.1001/jamasurg.2017.0898

[17] Pereira R J Munechika M Sakata R K Pain Management after Outpatient Surgical Procedure Rev Dor (São Paulo) 2013 14 01 61 67

[18] Feibel R J Dervin G F Kim P R Beaulé P E Major complications associated with femoral nerve catheters for knee arthroplasty: a word of caution J Arthroplasty 2009 24(6, Suppl)132 137 10.1016/j.arth.2009.04.008 19553071

[19] Ilfeld B M Duke K B Donohue M C The association between lower extremity continuous peripheral nerve blocks and patient falls after knee and hip arthroplasty Anesth Analg 2010 111 06 1552 1554 20889937 10.1213/ANE.0b013e3181fb9507 PMC3271722

[20] Muraskin S I Conrad B Zheng N Morey T E Enneking F K Falls associated with lower-extremity-nerve blocks: a pilot investigation of mechanisms Reg Anesth Pain Med 2007 32 01 67 72 10.1016/j.rapm.2006.08.013 17196495

[21] Jæger P Zaric D Fomsgaard J S Adductor canal block versus femoral nerve block for analgesia after total knee arthroplasty: a randomized, double-blind study Reg Anesth Pain Med 2013 38 06 526 532 24121608 10.1097/AAP.0000000000000015

[22] Sharma S Iorio R Specht L M Davies-Lepie S Healy W L Complications of femoral nerve block for total knee arthroplasty Clin Orthop Relat Res 2010 468 01 135 140 19680735 10.1007/s11999-009-1025-1 PMC2795813

[23] Jiang X Wang Q Q Wu C A Tian W Analgesic efficacy of adductor canal block in total knee arthroplasty: a meta-analysis and systematic review Orthop Surg 2016 8 03 294 300 27627711 10.1111/os.12268 PMC5129513

[24] Wang C G Ding Y L Wang Y Y Liu J Y Zhang Q Comparison of adductor canal block and femoral triangle block for total knee arthroplasty Clin J Pain 2020 36 07 558 561 32271182 10.1097/AJP.0000000000000833

[25] Gao F Ma J Sun W Guo W Li Z Wang W Adductor canal block versus femoral nerve block for analgesia after total knee arthroplasty: a systematic review and meta-analysis Clin J Pain 2017 33 04 356 368 27322397 10.1097/AJP.0000000000000402

[26] Goytizolo E A Lin Y Kim D H Addiction of adductor canal block to periarticular injection for total knee replacement J Bone Joint Surg Am 2019 101 09 812 820 10.2106/JBJS.18.00195 31045669

