# Supplementary material for: Periarticular Infiltration Compared to Single Femoral Nerve Block in Total Knee Arthroplasty: A Prospective Randomized Study
Source: Rev Bras Ortop (Sao Paulo). 2024 Apr 10;59(2):e241–6. doi: 10.1055/s-0044-1785449 (PMC11006530; doi:10.1055/s-0044-1785449)
Supplement: Related texts

# Research article: Infiltração periarticular comparada ao bloqueio do nervo femoral único na artroplastia total de joelho: Um estudo prospectivo randomizado

**Keywords:** analgesia, anestesia local, artroplastia do joelho, bloqueio nervoso, infiltração, nervo femoral

## Abstract

**Objetivo** Avaliar pacientes submetidos a artroplastia total do joelho (ATJ) sob raquianestesia e bloqueio do nervo femoral (BNF) único e comparar com pacientes que submetidos a ATJ sob raquianestesia e infiltração periarticular (IPA).

**Materiais e Métodos** Um total de 100 pacientes submetidos a ATJ foram randomizados em dois grupos. O grupo 1 incluiu pacientes submetidos a ATJ sob BNF associado à raquianestesia, ao passo que o grupo 2 incluiu pacientes submetidos a ATJ sob IPA associada à raquianestesia. Os indivíduos foram avaliados no pós-operatório precoce quanto à dor, à flexão e extensão ativas, à elevação do membro estendido e ao uso de morfina.

**Resultados** Não se observou diferença significativa associada ao tipo de analgesia em relação à dor, à elevação do membro em extensão e ao consumo de morfina. Houve melhor flexão e extensão ativas no grupo que recebeu IPA ( *p* = 0,04 e *p* = 0,02, respectivamente).

**Conclusão** Concluímos que as técnicas utilizadas são semelhantes quanto ao controle da dor, à elevação de membro e ao uso de morfina. O uso de IPA proporcionou uma melhor flexão e extensão ativas durante o período de internação hospitalar comparado ao uso de BNF único em pacientes submetidos a ATJ.

## Introdução

Mais de 80% dos pacientes submetidos a procedimentos cirúrgicos sentem dor aguda pós-operatória, e aproximadamente 75% destes a classificam como moderada ou severa. Evidências sugerem que o tratamento da dor pós-operatória é muitas vezes inadequado, com efeitos diretos na qualidade de vida, na recuperação e no risco de complicações pós-operatórias, como a dor crônica. 1 2 3 4

Entre os métodos de controle da dor após artroplastia total de joelho (ATJ), o bloqueio do nervo femoral (BNF) único com utilização de cateter e infusão anestésica intermitente proporciona boa analgesia pós-operatória, mas com a potencial desvantagem de diminuir a ativação do quadríceps, o que ocasiona menor controle muscular na marcha e risco de quedas. Estudos recentes 5 6 7 8 9 10 11 12 demonstraram que a infiltração periarticular (IPA) intraoperatória, utilizando um coquetel de medicamentos, produz efeito analgésico semelhante ao BNF, com a vantagem de não comprometer a função muscular.

O presente estudo tem como objetivo avaliar e comparar os pacientes submetidos a ATJ sob raquianestesia e BNF único com os pacientes submetidos ao mesmo procedimento, mas sob raquianestesia e IPA. Os pacientes foram avaliados no pós-operatório precoce quanto à dor, à amplitude de movimento (ADM) do joelho, capacidade de elevação do membro, necessidade do uso de morfina e presença de complicações. Nossa hipótese é a de que o uso da IPA produz o mesmo efeito analgésico no pós-operatório precoce de ATJ quando comparado ao BNF único, mas tem efeito menor sobre a função muscular na fase inicial da reabilitação.

## Materiais e Métodos

Ao todo, 100 pacientes com indicação de cirurgia de ATJ foram inicialmente selecionados e randomizados em dois grupos. O grupo 1 incluiu 50 pacientes submetidos a ATJ sob raquianestesia e BNF simples (único), ao passo que o grupo 2 foi formado por 50 pacientes submetidos ao mesmo procedimento cirúrgico, mas sob raquianestesia e IPA.

Os critérios de inclusão foram pacientes de ambos os sexos, com idade ≥ 18 anos, submetidos a ATJ primária por osteoartrose avançada. Foram excluídos pacientes com alergia a alguma das medicações inseridas no protocolo de pesquisa, com contraindicação ou falha da raquianestesia, com abuso conhecido de álcool ou drogas, portadores de artrite reumatoide ou outras doenças inflamatórias, pacientes submetidos a cirurgia prévia (exceto cirurgias para tratamento de lesões meniscais e ligamentares), aqueles com presença de doenças psiquiátricas diagnosticadas ou em tratamento prévio, com presença de plegia, paresia ou parestesia no membro contralateral, além de pacientes que não concordaram em participar da pesquisa.

Os participantes incluídos foram inicialmente randomizados em 2 grupos de interesse por meio de uma sequência de números aleatórios ordenados de 1 a 100, conforme a sua data de entrada no estudo. A lista de randomização foi mantida em sigilo por uma pessoa responsável, a qual informava ao cirurgião e ao anestesista, anteriormente à indução anestésica, em qual grupo o paciente se incluía. Essa mesma pessoa não participou de nenhuma outra fase do estudo, e não teve contato com os pacientes, nem com os avaliadores. A coleta de dados foi realizada por um médico integrante da equipe e devidamente treinado e cegado.

Os indivíduos do grupo 1 foram submetidos a raquianestesia com 15 mg de bupivacaína e 100 mcg de morfina e, logo após, a BNF único com 150 mg de ropivacaína e 150 mcg de clonidina, que foi realizado pelo anestesista com o auxílio de um estimulador de nervo periférico (Stimuplex, B. Braun Medical Inc., Melsungen, Hesse, Alemanha) . Os pacientes do grupo 2 receberam a combinação de raquianestesia, assim como o grupo 1, e IPA com solução analgésica composta de 150 mcg de clonidina, 30 mg de cetorolaco, 375 mg de ropivacaína e 1 mg de epinefrina diluídos em 50 mL de soro fisiológico. A IPA foi realizada pelo médico cirurgião durante o ato cirúrgico na distribuição de 20 mL na região capsular posteromedial, 15mL na região posterolateral, e o restante distribuído por toda a região subperiosteal femoral e tibial (lateral, medial e anterior).

A avaliação pós-operatória seguiu uma ordem lógica, e iniciou com a avaliação da dor, seguida pelas avaliações da ADM ativa e passiva, da elevação ativa do membro e da quantidade de morfina usada no pós-operatório. Os pacientes foram avaliados durante a internação com 24 (primeiro dia de pós-operatório [1PO]), 48 (2PO) e 72 (3PO) horas de pós-operatório. Todos seguiram o mesmo protocolo de fisioterapia pós-operatória e receberam o mesmo protocolo analgesia pós-operatória, o qual incluiu o uso contínuo de 30 mg de codeína a cada 6 horas, 750 mg de paracetamol a cada 8 horas, 1 g de dipirona a cada 6 horas e 30 mg de cetorolaco a cada 8 horas por 48 horas. A prescrição de morfina na dose de 0,05 mg/kg a cada 3 horas foi deixada a critério do paciente, conforme necessidade.

Para a avaliação da dor, foi usada a Escala Visual Analógica (EVA), em que o paciente indica um número de 0 a 10, sendo 0 sem dor e 10 a maior dor possível. As ADMs passiva e ativa do joelho foram avaliadas em graus com um goniômetro universal, com o paciente em decúbito dorsal. Quanto à elevação do membro estendido, considerou-se a distância em centímetros de elevação ativa do membro medida do calcâneo à mesa de exame físico. O uso de morfina foi quantificado em mg/dia e avaliado posteriormente em análise de prontuário.

O estudo transcorreu de setembro de 2019 a fevereiro de 2021, e todos os indivíduos foram atendidos e operados com o uso da mesma técnica cirúrgica e implantes. O procedimento anestésico foi realizado pela mesma equipe de anestesia. O estudo foi aprovado pela Comissão de Ensino e Pesquisa da Universidade de Passo Fundo em agosto de 2019 (sob o parecer 3.537.0920), e todos os indivíduos incluídos assinaram o termo de consentimento livre e esclarecido (TCLE).

## Análise Estatística

O cálculo do tamanho da amostra foi realizado no programa Windows Programs for Epidemiologists (Winpepi, *freeware* ), versão 11.65, e com base no estudo de Zhang et al. 7 Considerando um nível de significância de 5%, poder de 80% e um tamanho de efeito de no mínimo 0,6 desvios padrão (DPs) entre os grupos quanto à pontuação de dor, obteve-se um total mínimo de 44 sujeitos por grupo.

As variáveis quantitativas e ordinais foram descritas por valores de média ± DP, e as categóricas, por frequências absolutas e relativas. A normalidade dos dados foi avaliada pelo teste Shapiro-Wilk. Para comparar os grupos ao longo do tempo, foi utilizado o modelo de equações de estimativas generalizadas ( *generalized estimating equations* , GEE, em inglês) complementado pelo teste da diferença menos significativa ( *least significant difference,* LSD, em inglês). Para as variáveis com distribuição normal, o modelo linear foi aplicado, e para as variáveis com distribuição assimétrica ou ordinais, o modelo gama foi utilizado.

O nível de significância adotado foi de 5% ( *p* < 0,05), e as análises foram realizadas no programa IBM SPSS Statistics for Windows (IBM Corp., Armonk, NY, Estados Unidos), versão 28.0.

## Resultados

O estudo incluiu e analisou 100 pacientes, e a Tabela 1 contém a caracterização da amostra. Os grupos eram semelhantes em termos de idade, sexo e lado operado, e ambos tiveram média de internação de 4 dias.

**Tabela 1:** Caracterização da amostra

Variáveis	Amostra total (n = 100)	Grupo BNF (n = 50)	Grupo IPA (n = 50)	*p*
Idade (anos): média ± DP	67,9 ± 7,7	68,1 ± 7,5	67,7 ± 7,9	0,400*
Sexo: n (%)				1,000**
Masculino	23 (23,0)	11 (22,0)	12 (24,0)	
Feminino	77 (77,0)	39 (78,0)	38 (76,0)	
Local: n (%)				1,000**
Matriz	69 (69,0)	35 (70,0)	34 (68,0)	
Filial	31 (31,0)	15 (30,0)	16 (32,0)	
Lado: n (%)				0,316**
Direito	54 (54,0)	30 (60,0)	24 (48,0)	
Esquerdo	46 (46,0)	20 (40,0)	26 (52,0)	
Tempo de internação (dias): média ± DP	4,28 ± 0,52	4,39 ± 0,53	4,18 ± 0,48	0,208*

**Abreviaturas:** BNF, bloqueio do nervo femoral; DP, desvio padrão; IPA, infiltração periarticular.

**Notas:** *Teste *t* de Student; **Teste do qui-quadrado de Pearson.

Quanto à dor, não houve diferença estatisticamente significativa entre os grupos. O efeito de interação entre grupo e tempo quanto aos níveis de dor também não apresentou diferença. Em ambos os grupos, foi possível observar que o nível de dor no 3PO foi significativamente inferior ao dos outros dois dias, que não diferiram significativamente entre si. Mesmo após o ajuste pelo consumo diário de morfina, os resultados permaneceram semelhantes entre os grupos ( Tabela 2 ).

**Tabela 2:** Comparação entre os grupos das variáveis analisadas nos dias 1, 2 e 3 de pós-operatório

Variáveis	Grupo BNF (n = 50)	Grupo IPA (n = 50)	*p* ^#^
**DOR (EVA): média ± DP**			
1° dia de PO	4,94 ± 2,25 ^b^	4,94 ± 2,40 ^b^	1,000
2° dia de PO	4,86 ± 2,22 ^b^	4,32 ± 1,98 ^b^	0,195
3° dia de PO	2,84 ± 1,80 ^a^	2,88 ± 1,99 ^a^	0,915
Diferença entre o 1° e o 3° dias de PO (IC95%)	−2,10 (−2,68 a −1,52)	−2,06 (−2,69 a −1,43)	0,199**
**ADM ativa de flexão: média ± DP**			
1° dia de PO	42,7 ± 22,4 ^a^	47,9 ± 22,9 ^a^	0,248
2° dia de PO	56,2 ± 18,8 ^b^	63,4 ± 17,4 ^b^	**0,046**
3° dia de PO	77,8 ± 15,1 ^c^	83,0 ± 11,2 ^c^	**0,047**
Diferença entre o 1°e o 3° dias de PO (IC95%)	35,1 (29,5 a 40,6)	35,1 (29,8 a 40,4)	0,571**
**ADM ativa de extensão: média ± DP**			
1° dia de PO	1,30 ± 2,44 ^b^	1,22 ± 2,17 ^b^	0,869
2° dia de PO	1,10 ± 2,32 ^ab^	0,50 ± 1,52 ^a^	0,122
3° dia de PO	0,80 ± 1,85 ^a^	0,80 ± 1,85 ^ab^	1,000
Diferença entre o 1° e o 2° dias de PO (IC95%)	−0,20 (−0,47 a 0,07)	−0,72 (−1,21 a −0,24)	0,025**
Diferença entre o 2°e o 3° dias de PO (IC95%)	−0,30 (−0,73 a 0,13)	0,30 (−0,13 a 0,73)	
Diferença entre o 1° e o 3° dias de PO (IC95%)	−0,50 (−1,00 a −0,00)	−0,42 (−1,10 a 0,25)	
**Elevação (cm) – média ± DP**			
1° dia de PO	16,9 ± 21,8 ^a^	22,7 ± 25,4 ^a^	0,222
2° dia de PO	26,6 ± 24,2 ^b^	35,2 ± 25,0 ^b^	0,078
3° dia de PO	39,4 ± 23,6 ^c^	43,3 ± 23,7 ^c^	0,401
Diferença entre o 1° e o 3° dias de PO (IC95%)	22,5 (18,1 a 26,9)	20,7 (15,4 a 26,0)	0,160**
**Uso de morfina (mg)**			
1° dia de PO	3,62 ± 3,95 ^b^	4,20 ± 4,51 ^b^	0,489
2° dia de PO	2,71 ± 3,71 ^b^	2,95 ± 3,08 ^b^	0,722
3° dia de PO	0,59 ± 1,39 ^a^	1,16 ± 2,87 ^a^	0,201
Diferença entre o 1° e o −3° dias de PO (IC95%)	−3,03 (−4,02 a −2,04)	−3,04 (−4,48 a −1,60)	0,880**

**Abreviaturas:** ADM, amplitude de movimento; BNF, bloqueio do nervo femoral; DP, desvio padrão; EVA, Escala Visual Analógica; IC95%, intervalo de confiança de 95%; IPA, infiltração periarticular; PO, pós-operatório.

**Notas:** *+ = 1/++ = 2/+++ = 3/++++ = 4; **efeito de interação grupo *versus* tempo; ^a,b,c^ comparação intragrupo: letras iguais não diferem pelo teste da diferença menos significativa ( *least significant difference* , LSD, em inglês) a 5% de significância; ^#^ comparação entre os grupos por meio do teste LSD pelo modelo de equações de estimativas generalizadas ( *generalized estimating equations* , GEE, em inglês).

Houve diferença estatisticamente significativa entre os grupos quanto à ADM. Observou-se que o grupo IPA apresentou médias de flexão ativa significativamente mais elevadas no 2PO ( *p* = 0,046) e no 3PO ( *p* = 0,047), quando comparado ao grupo BNF ( Fig. 1 ). Quanto à extensão ativa, o comportamento ao longo do tempo foi significativamente diferente entre os grupos (efeito significativo de interação; *p* = 0,025): o grupo IPA teve uma redução significativa no 2PO (média: 0,72), e o grupo BNF teve uma redução significativa somente no 3PO ( Fig. 2 ). Entretanto, apesar de os dados apresentarem diferença estatística entre os grupos, observamos que a diferença encontrada pode não ser clinicamente relevante no médio e longo prazos.

**Fig. 1:** Avaliação da amplitude de movimento (ADM) ativa de flexão conforme o tempo e o grupo em estudo.

**Fig. 2:** Avaliação da ADM ativa de extensão conforme o tempo e o grupo em estudo.

Com relação aos parâmetros flexão ativa, contração do quadril e elevação, encontramos um aumento significativo a partir do 2PO em ambos os grupos. Quanto ao uso de morfina, somente houve uma redução significativa nos dois grupos no 3PO.

Dois pacientes (um de cada grupo) apresentaram reações alérgicas: prurido e exantema; e um paciente (do grupo BNF) sofreu queda ao solo da própria altura no 2PO, sem outras consequências.

## Discussão

Vários estudos demostraram que tanto o BNF quanto a IPA são técnicas confiáveis para o controle da dor no pós-operatório precoce de ATJ. 5 10 O controle inadequado da dor no período pós-operatório de ATJ aumenta o risco de dor crônica, com consequente piora da qualidade de vida, além de prolongar o tempo de internação hospitalar e elevar os custos do tratamento. 13 14 15

Este estudo buscou comparar o BNF único com a IPA no pós-operatório precoce de ATJ. Confirmou-se a hipótese de um controle adequado da dor associado a uma melhor capacidade de contração muscular no pós-operatório precoce no grupo IPA. O grupo BNF único apresentou efeito deletério na função muscular, representada pela flexão e extensão ativas durante a fase inicial da reabilitação, o que é sugerido por alguns autores. 5 7 16

A inervação sensitiva primária do joelho é suprida pelo nervo femoral anteriormente e pelo nervo cutâneo posterior da coxa posteriormente. O BNF único ou com analgesia contínua mediante o uso de cateter são métodos de analgesia pós-operatória capazes de proporcionar um melhor controle da dor e uma redução do consumo de opioides e de seus efeitos adversos. 12 Entretanto, é importante salientar que as técnicas anestésicas regionais requerem um especialista em anestesia, apresentam uma taxa de falha de 0% a 67%, e o risco de quedas devido à inibição motora tem sido relatado por alguns autores, 16 17 18 19 20 21 22 principalmente com o uso de bloqueios com liberação contínua de anestésico por cateter. Neste estudo, houve um relato de queda de um indivíduo do grupo de BNF no 2PO. O paciente não apresentava nenhuma limitação motora importante na avaliação, mas o fator causal do BNF não pode ser excluído.

A IPA ganhou espaço na prática clínica pelo fato de que vários estudos 7 8 9 11 12 mostram resultados equiparáveis ao BNF no controle da dor e no consumo de opioides. Albrecht et al., 5 em uma revisão sistemática de 14 ensaios clínicos randomizados, demonstraram alívio da dor e resultado funcional pós-operatório semelhante entre as técnicas de BNF e IPA. Wall et al. 10 randomizaram 230 pacientes com indicação de ATJ, e não houve diferença estatística entre os grupos submetidos a IPA e a BNF. Nesse estudo, 10 cabe destacar que as técnicas anestésicas foram realizadas por 59 anestesistas e 33 cirurgiões, o que representa grande variabilidade de execução, mas a reprodutibilidade dos resultados foi preservada.

Quanto ao consumo de morfina no período de internação hospitalar, alguns estudos prospectivos 7 8 12 não encontraram diferenças significativas. Por outro lado, Parvataneni et al. 11 encontraram dados divergentes em seu estudo: um maior consumo de morfina no 1PO pelos pacientes que foram submetidos a IPA. No presente estudo, não se observou diferença, pois os indivíduos de ambos os grupos apresentaram uma diminuição gradual no consumo de morfina ao longo dos dias.

Com relação à mobilidade do joelho, encontramos um ganho mobilidade ativa significativa nos indivíduos do grupo IPA. Berninger et al. 9 e Wall et al. 10 encontraram uma maior capacidade de flexão nos pacientes que receberam IPA, mais evidenciada no 1PO no primeiro estudo, e, no segundo estudo, no 2PO.

Apesar da diferença encontrada na mobilidade ativa, no presente estudo não foram encontradas diferenças significativas entre os grupos quanto à elevação do membro inferior. Acreditamos que a ausência de diferença pode estar associada à realização do BNF único, ou seja, sem infusão contínua de anestésico. Esse resultado diferiu dos apresentados por Parvataneni et al., 11 que encontraram no grupo IPA uma melhor capacidade de elevação do membro estendido no 1PO, com pontuações de dor semelhantes durante curso hospitalar pós-operatório, o que sugere que a IPA resulta em controle da dor equivalente ao do BNF, com a manutenção da força motora do quadríceps.

Recentemente, alguns estudos avaliaram o uso de bloqueio do canal do adutor (BCA) comparado com o uso do BNF, além do uso associado do BCA e da IPA. Comparado ao BNF, o BCA tem mostrado resultados semelhantes no controle da analgesia, mas com um menor envolvimento motor quando comparado ao BNF. 23 24 25 Quanto à associação do BCA e da IPA, Goytizolo et al. 26 não mostraram diferença na adição do BCA à IPA isolada; entretanto, novos estudos são necessários para elucidar essa questão.

Acreditamos que este estudo apresenta alguns pontos a serem observados. Todos os pacientes foram operados pela mesma equipe cirúrgica e anestésica, e receberam o mesmo protocolo da IPA, BNF e medicação analgésica. Procurando tornar o procedimento mais reprodutível em locais sem capacidade de realização do BNF assistido por ultrassonografia, optamos pela técnica de BNF único, uma vez que a técnica de infusão contínua requer treinamento específico e equipamento adequado. Escores funcionais de médio e longo prazo não foram utilizadas, tampouco escores de avaliação de qualidade de vida e saúde mental. Devemos salientar que a inexistência de estudos específicos com o mesmo grupo de pacientes na literatura limita a precisão do cálculo do tamanho da amostra. Nesse contexto, o cálculo do tamanho da amostra pode estar subdimensionado, pois foi usada como referência apenas a variável dor. Assim, mesmo que os resultados encontrados corroborem os existentes na literatura, os autores salientam a necessidade de estudos futuros para uma melhor padronização de procedimentos a respeito do tema, assim como a investigação do efeito da associação de procedimentos e a avaliação da relevância clínica dos achados.

## Conclusão

Concluímos que o controle da dor, a elevação de membro e o consumo de morfina foram semelhantes entre as técnicas de analgesia. A técnica de IPA proporciona uma maior capacidade de extensão e flexão ativas do joelho durante os três primeiros dias de pós-operatório.
